# Comprehensive profiling of phenotypic features of memory T- and B-cells from birth to the onset of old age

**DOI:** 10.3389/fragi.2026.1890974

**Published:** 2026-09-30

**Authors:** Joaquim Pedro Brito-de-Sousa, Laise Rodrigues Reis, Ismael Artur da Costa-Rocha, Vanessa Peruhype-Magalhães, Christiane Costa-Pereira, Julia Pereira Martins, Lis Ribeiro do Valle Antonelli, Cristiana Couto Garcia, Angelica Oliveira Gomes, Jordana Grazziela Alves Coelho-dos-Reis, Ana Maria de Caetano Faria, Andréa Teixeira-Carvalho, Ana Carolina Campi-Azevedo, Karen Cecília de Lima Torres, Olindo Assis Martins-Filho

**Affiliations:** 1 Programa de Pós-graduação em Imunologia e Parasitologia Aplicadas, Universidade Federal de Uberlândia, Uberlândia, Brazil; 2 Grupo Integrado de Pesquisas em Biomarcadores, Instituto René Rachou, Fundação Oswaldo Cruz (FIOCRUZ-Minas), Belo Horizonte, Brazil; 3 Florida Research and Innovation Center, Cleveland Clinic, Port Saint Lucie, FL, United States; 4 Laboratório de Biologia e Imunologia de Doenças Infecciosas e Parasitárias, Instituto René Rachou, Fundação Oswaldo Cruz (FIOCRUZ-Minas), Belo Horizonte, Brazil; 5 Departamento de Biologia Estrutural, Universidade Federal do Triângulo Mineiro, Uberaba, Brazil; 6 Departamento de Microbiologia, Instituto de Ciências Biológicas, Universidade Federal de Minas Gerais, Belo Horizonte, Brazil; 7 Departamento de Bioquímica e Imunologia, Instituto de Ciências Biológicas, Universidade Federal de Minas Gerais, Belo Horizonte, Brazil; 8 Universidade José do Rosário Vellano, UNIFENAS, Belo Horizonte, Brazil

**Keywords:** immunobiography, immunophenotyping, inflammaging, memory B-cells, memory T-cells

## Abstract

**Introduction:**

Human aging is accompanied by profound and continuous remodeling of the immune system, characterized by rhythmic, stage-specific patterns. These features are characterized by waves of cellular differentiation, network reorganization, and functional recalibration, a phenomenon encompassed by the concept of immunobiography.

**Methods:**

This study focuses on T- and B-cell memory immunophenotypes that shape adaptive immunity, including interferon-activated cells from healthy volunteers of both sexes, aged 9 months to 65 years, stratified into 12 consecutive age subgroups. Significant shifts were concentrated in intermediate age subgroups, particularly at 5–6 years, 10–19 years (the most pronounced), and 20–29 years.

**Results:**

Although women exhibited an earlier onset of changes, men demonstrated greater lifelong variability in differentiated subsets, especially for early effector CD4^+^ cells. Additionally, other cell subsets showed variability, especially in men, including early effector CD4^+^ and CD8^+^, interferon- mediated CD4^+^ and CD8^+^, central and effector memory CD4^+^, non-interferon-mediated CD4^+^, and naïve CD19^+^ B‐cells, predominantly between 10 and 39 years. Signature analyses confirmed sex-specific peaks occurring at distinct ages. Naïve cells displayed the highest overall connectivity, whereas B-cell network connectivity increased with age in women but decreased sharply in men after 20 years. Notably, the upregulation of immune memory cell subsets was accompanied by an increase in global connectivity.

**Discussion:**

Our findings indicate that immune memory remodeling across the human lifespan occurs in discrete, sex-dependent waves, probably shaped by cumulative antigen exposure and endocrine transitions. These trajectories may differentially balance adaptive immunity and inflammatory differentiation, potentially influencing long-term immune resilience and susceptibility to chronic inflammatory conditions.

## Introduction

Human aging is accompanied by profound and continuous remodeling of the immune system, tightly coupled to developmental and degenerative changes in endocrine function across the lifespan. From early life through puberty, adulthood, and senescence, immune cell composition and function are progressively shaped by cumulative antigen exposure, thereby shaping human adaptive immunity that is protective against infection hazards. In addition, with thymic involution, bone marrow remodeling, and the emergence of chronic low-grade inflammation, these processes collectively drive the transition from a naïve-dominant immune system to that increasingly enriched in memory, senescent, and pro-inflammatory cell populations (Li et al., 2019; Rodriguez et al., 2021; Kulesh et al., 2025). Superimposed on this antigen-driven immune trajectory, coordinated activity of the hypothalamic–pituitary–gonadal, hypothalamic–pituitary–adrenal, growth hormone, thyroid, and metabolic axes modulates adaptive immunity. Furthermore, theses axes coordinate changes associated to immune differentiation, memory, and functional polarization across distinct ages that fine-tune the immune response to accomplish developmental and physiological demands (Mauras et al., 1996; Livadas and Chrousos, 2019; Martín et al., 2021; Upadhyay et al., 2025).

The adaptive immune response has become a priority area for researching the aging process and its implications (Nikolich-Žugich, 2018; Goronzy and Weyand, 2017). Aging-associated changes in the immune system are clinically relevant, and T- and B-cell aging play a pivotal role in rendering older subjects vulnerable to infections, oncological and immune-mediated diseases, especially due to impaired memory responses. Examining T- and B-cell phenotypic changes along aging provided opportunities to examine major pathways and their consequences for memory cell functions. In addition, studies on T- and B-cell memory offer a unique opportunity to provide evidence to further explore the mechanisms driving the aging process in general (López-Otín et al., 2013). The T- and B-cell compartments are, therefore, appropriate fields to study the influence of aging on the dynamic rhythms of adaptive immune memory comparing distinct cell phenotypes (Yanes et al., 2017). The T-cell compartment has intrinsic mechanisms of remodeling, with the production of naïve T-cells dependent on thymic activity, which declines from adolescence to adulthood (Palmer, 2013). It has been widely demonstrated that aging has a strong impact on the remodeling of the B-cell compartment. Several pieces of evidence demonstrated a significant decrease in circulating B-cells early in childhood and a shift from naïve to memory cell phenotypes, accompanied by the impaired ability to produce antibodies to new antigens. Indeed, in the elderly, there is a reduction in naïve B-cells, accompanied by the expansion of memory B-cells that show a senescence-associated phenotype (Bulati et al., 2017). Overall, age-related impairment of T- and B-cell compartments certainly contributed to the development of age-related diseases.

Importantly, it has been postulated that immune remodeling across the lifespan does not occur as a linear or uniform process but rather follows rhythmic, stage-specific waves of cellular differentiation, network reorganization, and functional recalibration. This dynamic phenomenon is conceptualized immunobiography (Simon et al., 2015; Franceschi et al., 2017). During childhood and adolescence, successive developmental windows are marked by discrete shifts in the balance between naïve and memory T- and B-cell subsets that compose adaptive immunity, reflecting the cumulative impact of antigenic challenges early in life due to infections, vaccinations, and microbiome exposure (Saule et al., 2006; Ciocca et al., 2021; Brito-de-Sousa et al., 2023).

These early changes in immune cellularity are not transient but reverberate later in life, shaping the architecture, responsiveness, and inflammatory tone of the adult and aging immune system. In this perspective, aging can be viewed as a continuum that begins at birth, in which developmental immune adaptations during childhood and adolescence lay the foundation for immune resilience, strengthening resistance to damage and infections, susceptibility to chronic inflammation, and the risk of immune-mediated diseases in adulthood and advanced age (Franceschi et al., 2017; Brito-de-Sousa et al., 2023). Thus, lifespan-associated immune remodeling emerges from the dynamic integration of endocrine maturation, antigenic experience, and evolving cellular networks rather than from a simple accumulation of age-related defects. In this line, the present study aimed at characterizing the dynamic of changes in phenotypic features within memory T- and B-cell compartments along aging from birth to the onset of old age. Our hypothesis is that memory T and B-cells present a non-linear rhythmic profile during human development and aging, reflecting a remodeling process with distinct kinetics between men and women, which diverge primarily after the onset of gonadarche and puberty. Overall, these changes throughout lifetime define the human immunobiography.

## Population, material, and methods

### Study population and ethical statements

This study was designed as a descriptive-observational investigation carried out in the Southeast and Northeast regions of Brazil from 1 March 2011 to 30 November 2019. The present investigation comprised a non-probabilistic sample of 1,796 volunteers of both sexes, including 926 female and 870 male individuals, aged 9 months to 65 years.

Participants were recruited when they presented for routine vaccination at primary care medical centers. Inclusion criteria included healthy volunteers of both sexes with age ranging from 9 months to 65 years. Exclusion criteria included subjects with vaccination records within 30 days prior blood collection, pregnant women, HIV carriers, patients with chronic immune-mediated diseases, and patients receiving immunosuppressive therapy. Moreover, patients with hemoglobinopathies, history of blood transfusion, or previous treatment with hyperimmune serum up to 90 days prior to blood collection were not included in the present study.

The study population was initially clustered into 12 subgroups comprising consecutive age ranges, referred to as <1 year (n = 208), 1 year (n = 50), 2 years (n = 189), 3 years (n = 148), 4 years (n = 71), 5–6 years (n = 164), 7–9 years (n = 209), 10–19 years (n = 320), 20–29 years (n = 122), 30–39 years (n = 169), 40–49 years (n = 99), and 50–65 years (n = 47). Subsequently, the participants were categorized according to sex, following the same age stratification, as follows: <1 year (n = 112; 96), 1 year (n = 29; 21), 2 years (n = 100; 89), 3 years (n = 69; 79), 4 years (n = 25; 46), 5–6 years (n = 83; 81), 7–9 years (n = 104; 105), 10–19 years (n = 111; 209), 20–29 years (n = 98; 24), 30–39 years (n = 102; 67), 40–49 years (n = 60; 39), and 50–65 years (n = 33; 14) (female; male, respectively). Table 1 summarizes the demographic features of the study population stratified by sex.

**Table 1:** Demographic features of the study population stratified by age range.

Age Ranges	Study Population (n); Median Age (IQR)	Females (n); Median Age (IQR)	Males (n); Median Age (IQR)
<1 Year	208; 0.9 (0.9–0.9)	112; 0.9 (0.9–0.9)	96; 0.9 (0.9–0.9)
1 Year	50; 1.2 (1.0–1.3)	29; 1.1 (1.1–1.3)	21; 1.2 (1.0–1.3)
2 Years	189; 2.0 (1.8–2.2)	100; 1.9 (1.8–2.1)	89; 2.0 (1.8–2.2)
3 Years	148; 3.0 (2.8–3.1)	69; 2.9 (2.8–3.1)	79; 3.0 (2.8–3.1)
4 Years	71; 4.1 (3.8–4.3)	25; 4.2 (3.9–4.3)	46; 4.0 (3.8–4.3)
5–6 Years	164; 5.1 (4.9–5.4)	83; 5.1 (4.9–5.6)	81; 5.1 (4.9–5.3)
7–9 Years	209; 8.1 (7.9–8.4)	104; 8.1 (7.9–8.6)	105; 8.0 (7.8–8.4)
10–19 Years	320; 11 (11–18)	111; 11 (11–12)	209; 18 (11–18)
20–29 Years	122; 25 (23–28)	98; 25 (23–28)	24; 25 (21–27)
30–39 Years	169; 34 (31–37)	102; 34 (31–37)	67; 35 (33–37)
40–49 Years	99; 44 (42–46)	60; 45 (42–47)	39; 43 (41–45)
50–65 Years	47; 52 (51–54)	33; 52 (51–54)	14; 53 (50–60)
Total	1,796	926	870

Data are presented as the number of participants (n) and median age with interquartile range (IQR) for the overall study population and according to sex.

The study protocol adhered to the principles of the Declaration of Helsinki and the World Medical Association’s Code of Ethics for human research. The research protocol was submitted to and approved by the Ethics Committee for Studies with Humans at Instituto René Rachou, Fiocruz-Minas (CAAE: 0023.0.245.000–10, approval number: 23.2010 from 28 February 2011; CAAE: 25315213.6.0000.5091, approval number: 1.395.368 from 26 January 2016; and CAAE: 51160215.7.0000.5091, approval number: 2.401.123 from 27 November 2017). All participants or their next of kin have read and signed the informed consent prior enrollment. A compendium of study population and methods is presented in Figure 1.

**FIGURE 1:**
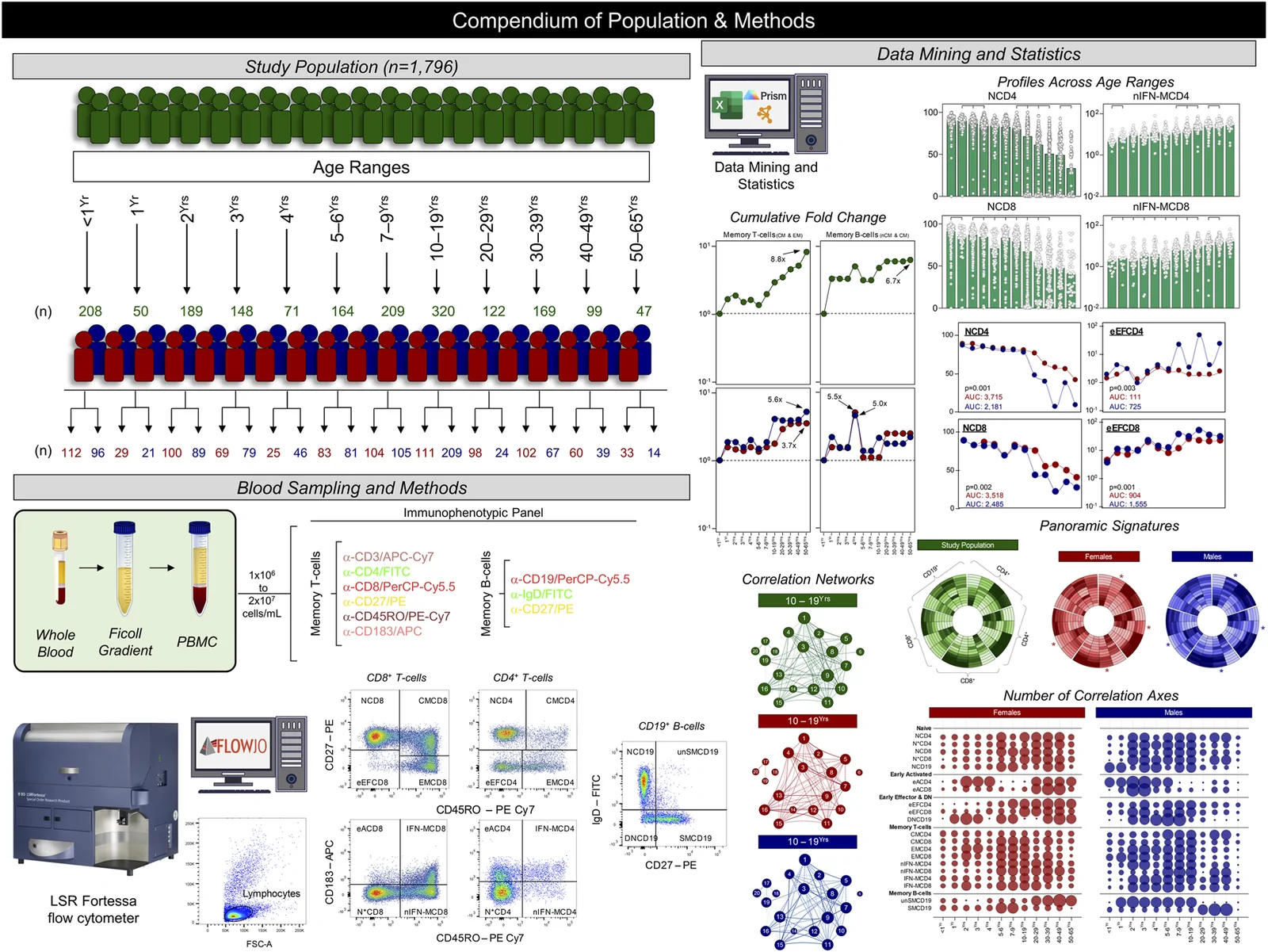
Compendium of study design, population, and methods. The study population comprised 1,796 individuals aged between 9 months and 65 years. The participants were categorized into distinct age ranges, such as <1 year, 1 year, 2 years, 3 years, 4 years, 5–6 years, 7–9 years, 10–19 years, 20–29 years, 30–39 years, 40–49 years, and 50–65 years. Blood samples collected were used to obtain PBMC that was stored at −80 °C until use for immunophenotypic analysis of memory T-cell and B-cell subsets. Standard operational procedures were carried out according to good laboratory practices, as described in the *Materials and methods* section. Sample acquisition was performed using DIVA software in a LSRFortessa flow cytometer, and data were analyzed using FlowJo software. Frequencies of memory T- and B-cell subsets were determined using distinct gating strategies. Distinct approaches were employed for data mining and statistics, including comparative analyses of memory T- and B-cell subset profiles across age ranges and sexes, cumulative fold change across age ranges, panoramic signatures, integrative networks, and axes of connections.

### Biological samples and mononuclear cell isolation

Whole peripheral blood samples (7–20 mL) were collected from each participant at primary care medical centers after pre-screening for routine vaccination by a trained nurse. Blood samples were collected by venipuncture using a vacuum system with gel separation without anticoagulants and were used to isolate peripheral blood mononuclear cells (PBMCs), as previously described by Reis et al. (2022). In brief, PBMCs were isolated using Histopaque-1077 density-gradient centrifugation at 500 x g for 40 min at room temperature. The PBMC layer was collected and washed with cold RPMI-1640. Thereafter, the cells were counted and resuspended to a final concentration of 1 × 10^6^ to 2 × 10^7^ cells/mL for immunophenotypic analysis.

### Flow cytometric immunophenotypic analysis of memory T- and B-cells

The immunophenotypic analysis of memory T- and B-cell subsets was carried out as previously described by Campi-Azevedo et al. (2019). In brief, aliquots of 5.0 × 10^5^ cells/well were incubated with RPMI-1640 medium supplemented with 5% heat-inactivated normal human AB serum, 2 mM L-glutamine, and 3% antibiotic/antimycotic at 37 °C in a 5% CO_2_ atmosphere for 144 h. Subsequently, cells were harvested from the plates and washed once with FACS buffer (phosphate buffered saline supplemented with 0.5% bovine serum albumin) before being stained with a mixture of monoclonal antibodies (mAbs) against cell-surface molecules to identify memory T-cell subsets (anti-CD3/SK7/APC-Cy7, anti-CD4/RPA-T4/FITC, anti-CD8/SK1/PerCP-Cy5.5, anti-CD45RO/UCHL1/PE-Cy7, anti-CD27/M-T271/PE, and anti-CD183/1C6/APC), and memory B-cell subsets (anti-CD19/HIB19/PerCP, anti-CD27/M-T271/PE, and anti-IgD/IA6-2/FITC), all purchased from BD Biosciences (San Jose, CA, USA). After incubation for 30 min at room temperature, cells were washed with cold FACS buffer and fixed with FACS fix solution (10.0 g/L paraformaldehyde, 10.2 g/L sodium cacodylate, and 6.65 g/L sodium chloride, all purchased from Sigma, St. Louis, MO, USA). At least 100,000 lymphocytes/sample were acquired using an LSRFortessa Flow Cytometer (BD Bioscience, San Diego, CA, USA). FlowJo software (version 10.8, BD Bioscience, San Diego, CA, USA) was used to quantify the frequency of memory T- and B-cell subsets, using distinct gating strategies as previously described (Reis et al., 2022; Campi-Azevedo et al., 2019; Marín et al., 2012; Moura et al., 2017; Costa-Pereira et al., 2018; Castlem et al., 2023). A number of memory T-cell phenotypic features was evaluated, including naïve (N = CD27^+^CD45RO^−^), early effector (eEF = CD27^−^CD45RO^−^), central memory (CM = CD27^+^CD45RO^+^), effector memory (EM = CD27^−^CD45RO^+^), naïve* (N* = CD183^−^CD45RO^−^), early activated (eA = CD183^+^CD45RO^−^), non-interferon-mediated (nIFN-M = CD183^−^CD45RO^+^), and interferon-mediated (IFN-M = CD183^+^CD45RO^+^) within CD4^+^ and CD8^+^ T-cells. B-cell analysis included naïve (N = CD19^+^CD27^−^IgD^+^), double negative (DN = CD19^+^CD27^−^IgD^-^), unswitched memory (unSM = CD19^+^CD27^+^IgD^+^), and class-switched memory (SM = CD19^+^CD27^+^IgD^−^). The results were expressed as the percentage of each memory phenotype within gated cells. Gating strategies are shown in Figure 2.

**FIGURE 2:**
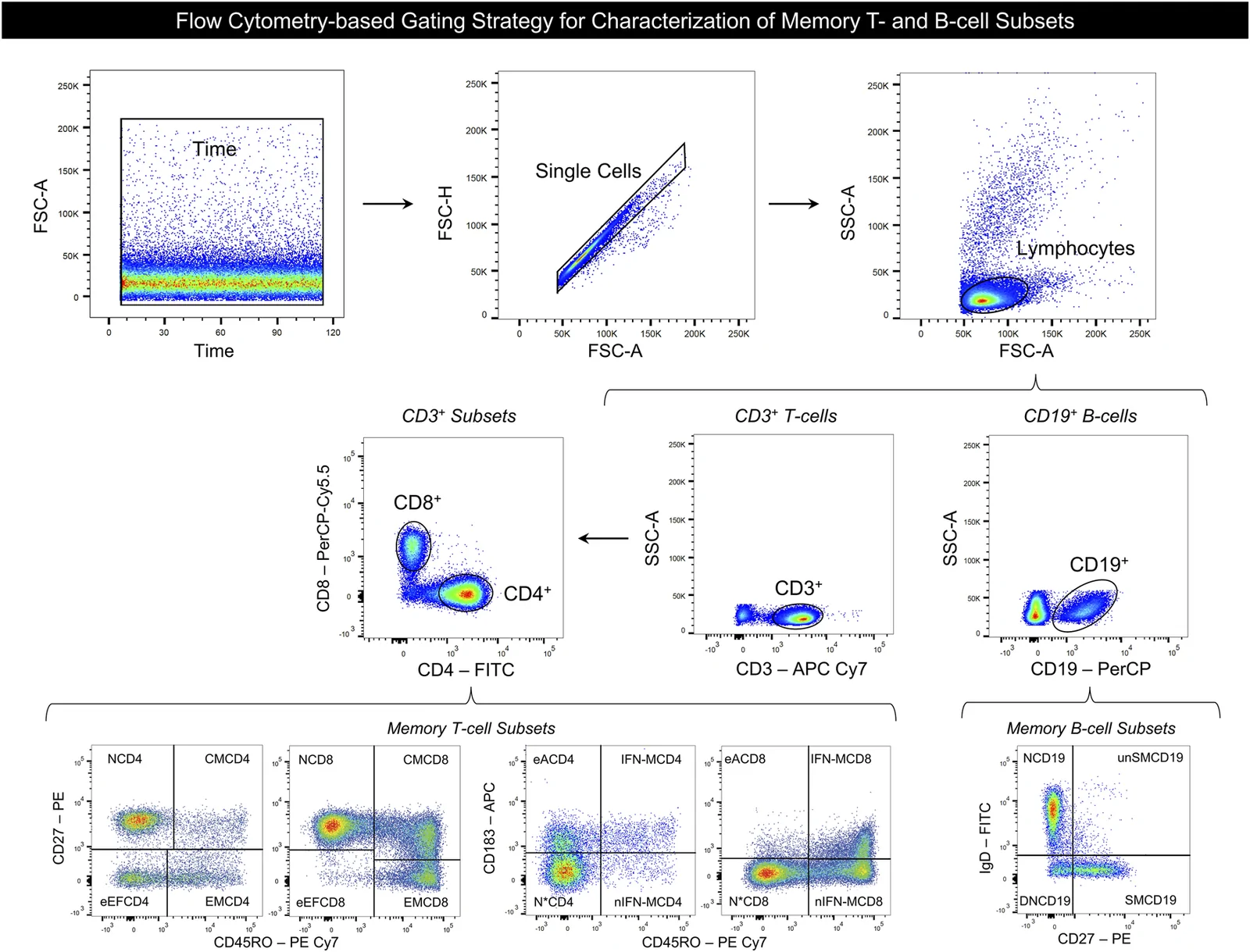
Representative flow cytometry gating strategies for phenotypic analysis of memory T-cells and B-cells. Immunophenotypic staining of PBMC were carried out using flow cytometry, as described in the *Materials and methods* section. Data acquisition was carried out using an LSRFortessa Flow Cytometer, with a total of 100,000 lymphocytes acquired per sample. FlowJo™ Software (version 10.7.2) was used for the analysis of flow cytometry data. Representative pseudocolor density plots illustrate the gating strategies used to analyze memory T- and B-cell subsets. Gating strategies start with event selection using time vs. side scatter area (SSC-A) to assess the acquisition quality of the continuous laser scatter profile. Subsequently, single cells were gated based on the forward scatter area (FSC-A) vs. forward scatter height (FSC-H) parameters. Thereafter, lymphocytes were selected based on their size vs. granularity morphometric properties (FSC-A vs. SSC-A). Phenotypic analysis of memory T- and B-cell subsets were further performed using bidimensional fluorescence pseudocolor density plots. The quantification of memory CD4^+^ and CD8^+^ T-cell subsets were carried out according to the CD45RO and CD27 immunostaining to identify naïve (N, CD45RO^−^CD27^+^), early effector (eEF, CD45RO^−^CD27^−^), central memory (CM, CD45RO^+^CD27^+^), and effector memory (EM, CD45RO^+^CD27^−^). Quantification of interferon-activated memory T-lymphocyte subpopulations was performed using CD45RO and CD183 immunostaining to identify naïve* (N*, CD183^−^CD45RO^−^), early activated (eA, CD183^+^CD45RO^−^), non-interferon-mediated (nIFN-M, CD183^−^CD45RO^+^), and interferon-mediated (IFN-M, CD183^+^CD45RO^+^) T‐cells. The analysis of memory B-cell subsets were performed based on the immunostaining profile of IgD and CD27 to identify naïve (N, CD27^−^IgD^+^), double-negative (DN, CD27^−^IgD^−^), unswitched memory (unSM, CD27^+^IgD^+^), and class-switched memory (SM, CD27^+^IgD^−^).

### Statistical analysis

Multiple strategies were applied for data mining and statistics. GraphPad Prism v9.0.1 software (San Diego, USA) was used for all statistical analyses, considering a threshold of p < 0.05 to define statistical significance.

Formal statistical analysis was first performed to define the normality distribution of datasets. For this purpose, the Shapiro–Wilk and Kolmogorov–Smirnov tests were used. Considering the non-parametric distribution of datasets, comparative analysis between pairs of adjacent age ranges and comparisons between sexes at matching age ranges were performed using the Mann–Whitney test.

Changes in memory T- and B-cell subsets according to age were further assessed after data categorization using cut-offs established at the 50th percentile of the empirical distribution of each dataset. The logistic regression model was used to quantify the association between the frequencies of memory T- and B-cell subsets and age continuum for putative variations in the study population and sex-stratified subgroups, considering a significance level of 5%. Based on the adjusted model, the predicted probabilities of subjects to present a given memory phenotype were estimated according to age continuum (years). The analysis was performed using R software (version 4.6.1), with the base stats package (glm version 4.4.1), and graphical outputs were generated using the ggplot2 package (4.0.3).

Analyses of rhythmic profiles of memory cell subsets across aging were performed using the area under the curve (AUC) as a summary measure. Comparative analysis between AUC from “Females” vs. “Males” was carried out using the Wilcoxon paired test. Additionally, age-related changes in memory cell subsets were assessed by calculating the cumulative fold changes across aging, calculated from successive ratios with significant differences in the preceding Mann–Whitney tests.

Panoramic signatures of memory T- and B-cell subsets were constructed as previously described by Luiza-Silva et al. (2011). For this purpose, the original data expressed as continuous variables (percentage of gated cells) were converted into categorical data (proportion) using the global median values established for each memory T- and B-cell subsets. The global median values were used to estimate the proportion of subjects above each cut-off edge. Descriptive analysis of signature profiles was first performed by considering the age range with the highest frequencies (peak) of memory T- and B-cell subsets. Thereafter, comparative analysis between the signatures of “Females” vs. “Males” was performed using the Fisher exact test. The analysis of delta signatures between sexes (delta = %Female − %Male) was further used for comparative analysis.

Integrative network structures were generated to compile significant correlations calculated using the Spearman rank test. The Spearman “r” scores were classified according to Akoglu (2018) and used to construct networks using the system biology approaches of the Cytoscape (Version 3.10.3) open-source platform (available at https://cytoscape.org). The category cluster layout was chosen for arranging the networks of memory T-cell and B-cell subsets, with nodes representing each memory subset and connecting lines identifying positive (“r” scores >0, continuous line) or negative (“r” scores <0, dashed line) correlations. The node sizes are proportional to the number of correlations between pairs of memory subsets. Venn diagram analysis was performed to identify common (shared) correlation axes between adjacent age ranges. Descriptive analysis of the integrative networks was carried out by considering the total number of correlations and the number of correlations identified for categories of memory subsets (naïve, memory T-cells, and memory B-cells). Line charts were assembled to characterize the profile of correlation numbers between memory subsets across aging. Colormap analysis was used to illustrate the differences between sexes, using color key based on the percentile distribution of each dataset (10th/50th/90th).

## Results

### Age-related remodeling of memory T- and B-cell subsets from birth to the onset of old age

Analysis of memory T- and B-cell subsets was performed to characterize the general profile of healthy individuals categorized into distinct and consecutive age-range subgroups (Figure 3). Data demonstrated that from <1 year to 1 year, eEFCD4, EMCD4, nIFN-MCD4, eEFCD8, unSMCD19, and SMCD19 levels were increased, accompanied by reductions in N*CD4, NCD8, and NCD19. Fewer changes occurred between 1 year and 2 years, featured by an increase in EMCD4 and decreases in NCD4 and CMCD8. From this point onward, wavering changes were observed between adjacent age ranges. Between 2 years and 3 years, increases were observed in CMCD4, EMCD4, IFN-MCD4, CMCD8, EMCD8, and IFN-MCD8, alongside decreases in CD45RO^−^ subsets, including NCD4, eEFCD4, N*CD4, and NCD8. During the transition from 3 years to 4 years, eEFCD4, EMCD4, eEFCD8, CMCD8, EMCD8, nIFN-MCD8, IFN-MCD8, DNCD19, and unSMCD19 were increased, with concomitant decreases in NCD4 and NCD8. The shift from 4 years to 5–6 years was marked by decreases in activated and memory subsets across T- and B-cell subsets, along with increases in eEFCD4, N*CD4, NCD8, and NCD19. This pattern was followed by the expansion of CMCD4, EMCD4, CMCD8, EMCD8, nIFN-MCD8, and DNCD19 between 5–6 years and 7–9 years, while some naïve and early activated subsets decreased (Figure 3).

**FIGURE 3:**
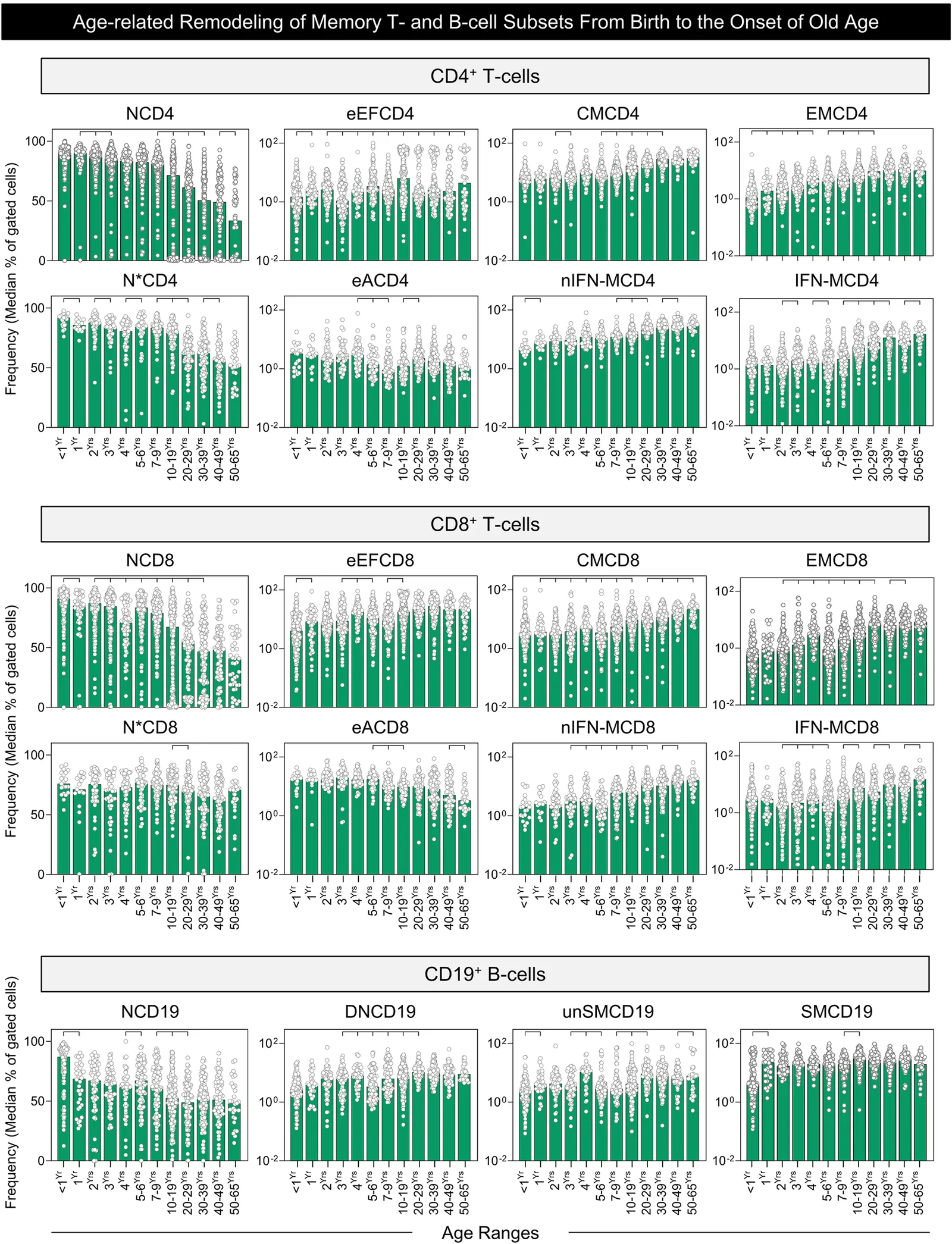
Age-related remodeling of memory T- and B-cell subsets from birth to the onset of old age. The overall profile of memory T- (CD4^+^ and CD8^+^) and B-cell subsets was characterized in peripheral blood samples from healthy individuals (study population/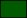, n = 1,796), categorized into distinct age ranges (<1 year, n = 208; 1 year, n = 50; 2 years, n = 189; 3 years, n = 148; 4 years, n = 71; 5–6 years, n = 164; 7–9 years, n = 209; 10–19 years, n = 320; 20–29 years, n = 122; 30–39 years, n = 169; 40–49 years, n = 99; and 50–65 years, n = 47). Immunophenotypic analyses of naïve (N), early effector (eEF), central memory (CM), effector memory (EM), naïve* (N*), early activated (eA), non-interferon-mediated (nIFN-M), and interferon-mediated (IFN-M) T-cells, as well as naïve (N), double-negative (DN), unswitched memory (unSM), and class-switched memory (SM) B cells were carried out using flow cytometry, as described in the *Materials and methods* section. The results are shown as the scattering distribution of individual data (% of gated cells) over bars of median values for each age range. Comparative analysis between pairs of adjacent age ranges was carried out using the Mann–Whitney test, with significant differences at p < 0.05 underscored by connecting lines for comparisons with the immediately earlier age range.

The transition from 7–9 years to 10–19 years represented one of the most pronounced remodeling phases, driven by increases across memory and interferon-mediated memory T-cell subsets, as well as in the B-cell compartment, combined with decreases in naïve subsets. Between 10–19 years and 20–29 years, immune differentiation progressed further, accompanied by the expansion of central, effector, and IFN-mediated CD4^+^ and CD8^+^ memory T-cells, along with increases in DNCD19 and unSMCD19, and continued reduction in naïve subsets (Figure 3).

Beyond 20–29 years, fewer age-related changes become noticeable. Between 20–29 years and 30–39 years, increases in eEFCD4, CMCD4, IFN-MCD4, CMCD8, and IFN-MCD8 were observed, alongside reductions in naïve CD4^+^ and CD8^+^ T-cells. From 30–39 years to 40–49 years, changes were more restricted involving IFN-mediated memory T-cell subsets within the CD8^+^ subset, together with decreases in eEFCD4 and N*CD4. Between 40–49 years and 50–65 years, increases in eEFCD4, IFN-MCD4, CMCD8, IFN-MCD8, and unSMCD19 were noticed, coupled with decreases in NCD4 and eACD8 (Figure 3).

### Age-related remodeling of memory T- and B-cell subsets from birth to the onset of old age according to sex

To better characterize the dynamic profile of changes in memory T- and B-cell subsets according to sex, the study population was categorized into female and males subgroups according to age ranges (Figure 4).

**FIGURE 4:**
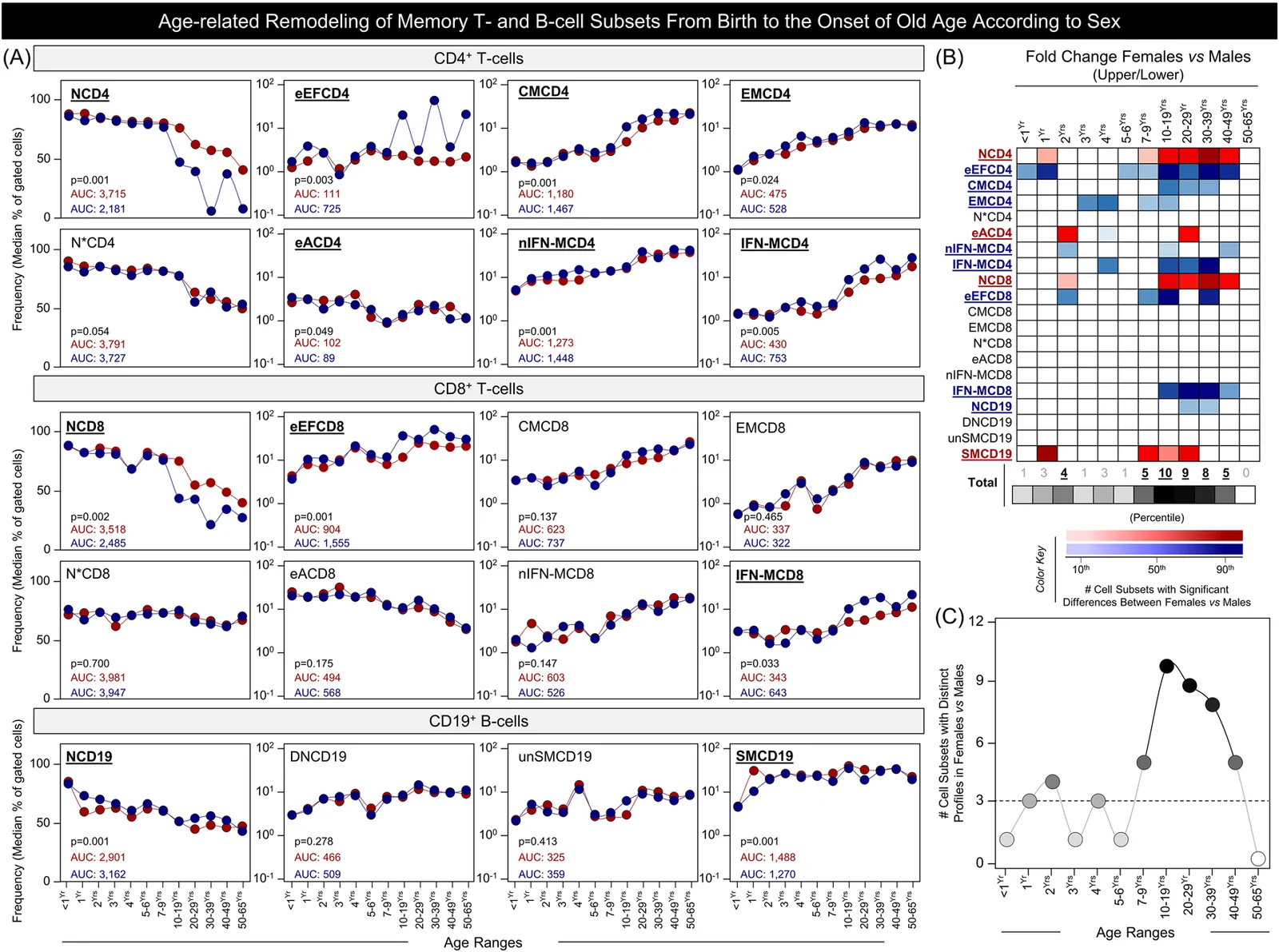
Age-related remodeling of memory T- and B-cell subsets from birth to the onset of old age according to sex. The overall profile of memory T- (CD4^+^ and CD8^+^) and B-cell subsets was characterized in peripheral blood samples from healthy individuals (n = 1,796), categorized according to sex (female/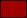, n = 926; male/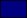, n = 870) and followed by stratification into distinct age ranges (<1 year, n = 119, n = 96; 1 year, n = 29, n = 21; 2 years, n = 100, n = 89; 3 years, n = 69, n = 79; 4 years, n = 25, n = 46; 5–6 years, n = 83, n = 81; 7–9 years, n = 104, n = 105; 10–19 years, n = 111, n = 209; 20–29 years, n = 98, n = 24; 30–39 years, n = 102, n = 67; 40–49 years, n = 60, n = 39; and 50–65 years, n = 33, n = 14, respectively). Immunophenotypic analyses of naïve (N), early effector (eEF), central memory (CM), effector memory (EM), naïve* (N*), early activated (eA), non-interferon-mediated (nIFN-M), and interferon-mediated (IFN-M) T-cells, as well as naïve (N), double-negative (DN), unswitched memory (unSM), and class-switched memory (SM) B-cells were carried out using flow cytometry, as described in the *Materials and methods*. **(A)** The results are shown as the line plot of the median frequencies values (% of gated cells) for each age range. Comparative analysis of the area under the curve (AUC) was performed using the Wilcoxon test. **(B)** The Mann–Whitney test was used to compare pairs of adjacent age ranges and differences between sexes. Fold change was calculated as the ratio of the larger value to the smaller value and was visualized using a color map, and **(C)** the total number of cell subsets with distinct profiles is presented as a line graph. The color keys are provided in the figure. In all statistical analyses, a difference with p < 0.05 was considered statistically significant.

The results demonstrated differences between female and male subgroups in 12 cell subsets. Overall, the female subgroup exhibited larger areas under the curve for NCD4, eACD4, NCD8, and SMCD19 than the male subgroup. In contrast, the male subgroup displayed larger areas under the curve for eEFCD4, CMCD4, EMCD4, nIFN-MCD4, IFN-MCD4, eEFCD8, IFN-MCD8, and NCD19 than the female subgroup (Figures 4A,B).

The summary of these results suggested that most differences between female and male subgroups occurred between 7–9 years and 40–49 years, with changes being higher predominantly in the male subgroup, peaking between 10–19 years (Figures 4B,C).

### Cumulative fold change between adjacent age ranges from birth to the onset of old age according to sex

The analyses of cumulative fold change across the lifespan provide novel insights regarding the remodeling of memory T and B-cells along aging (Figure 5). Data analysis showed that in general, two major patterns emerged: a pronounced decrease in naïve and early activated subsets and a considerable expansion of early effector and double-negative cells, along with memory T- and B-cell subsets. Of note, the magnitude of changes and the age of onset differ according to sex (Figure 5).

**FIGURE 5:**
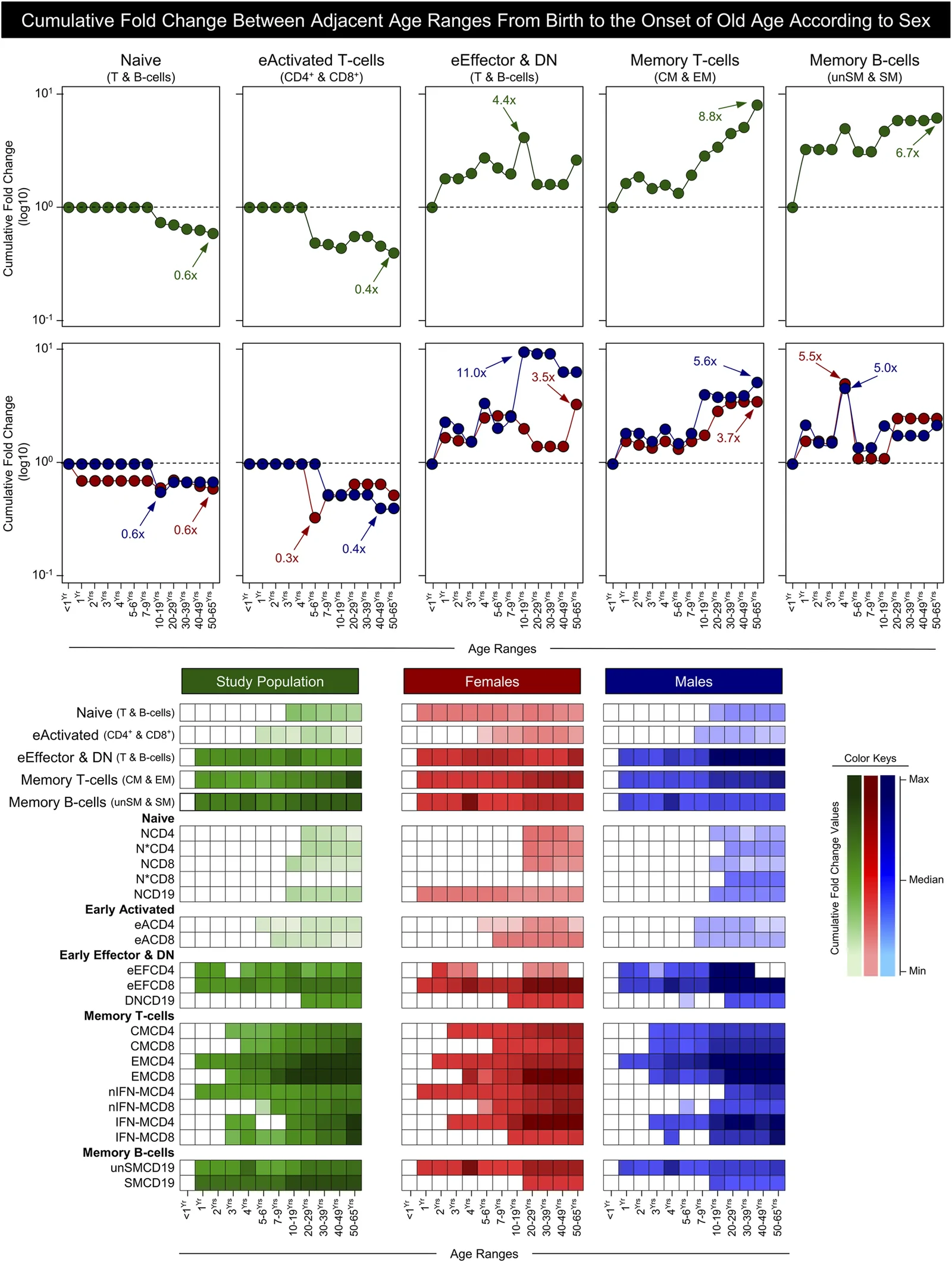
Cumulative fold change between adjacent age ranges from birth to the onset of old age according to sex. The overall profile of memory T-cell (CD4^+^ and CD8^+^) and B-cell subsets was characterized in PBMC from healthy individuals (study population/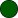, n = 1,796), categorized according to sex (female/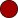, n = 926; male/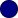, n = 870) and followed by stratification into distinct age ranges (<1 year, n = 119, n = 96; 1 year, n = 29, n = 21; 2 years, n = 100, n = 89; 3 years, n = 69, n = 79; 4 years, n = 25, n = 46; 5–6 years, n = 83, n = 81; 7–9 years, n = 104, n = 105; 10–19 years, n = 111, n = 209; 20–29 years, n = 98, n = 24; 30–39 years, n = 102, n = 67; 40–49 years, n = 60, n = 39; and 50–65 years, n = 33, n = 14, respectively). Immunophenotypic analyses of naïve (N), early effector (eEF), central memory (CM), effector memory (EM), naïve* (N*), early activated (eA), non-interferon-mediated (nIFN-M), and interferon-mediated (IFN-M) T-cells, as well as naïve (N), double-negative (DN), unswitched memory (unSM), and class-switched memory (SM) B-cells were carried out using flow cytometry, as described in the *Materials and methods* section. Cumulative fold changes across aging were calculated from successive ratios with significant differences in the preceding Mann–Whitney tests. The results are presented as line plots on a log scale with the peak marked in each graph. Color maps are constructed to display the individual values for each group in each age range. The color keys are provided in the figure.

In naïve subsets, the changes start as early as 1 year in female individuals, while shifts in male subjects occurred at 10–19 years. Early activated T-cell subsets also presented earlier decrease in females than in male subjects. Conversely, early effector and memory T-cell subsets presented an earlier onset in the male subgroup, while the magnitude of changes in memory B-cell subsets was greater in female individuals, especially from 20–29 years onward (Figure 5).

Taken together, despite the common reduction in naïve and early activated subsets coupled with the increase in memory cell subsets, the transitions appear to be earlier in the female subgroup, except for early effector and double-negative cells that displayed more prominent changes in male subjects (Figure 5).

### Predicted probability of changes in memory T- and B-cell subsets from birth to the onset of old age and according to sex

The predicted probabilities of subjects to present a given memory phenotype were calculated by logistic regression to assess the association between memory T- and B-cell subsets with age continuum (Figure 6). Data analysis showed that naive T- and B-cells (NCD4, N*CD4, NCD8, N*CD8, and NCD19) presented a continuous decrease in values along aging. A decrease in eACD8 and increases in eEFCD8 and DNCD19 were associated with age continuum. Additionally, memory T- and B-cell subsets (CMCD4, EMCD4, nIFN-MCD4, IFN-MCD4, CMCD8, EMCD8, nIFN-MCD8, IFN-MCD8, unSMCD19, and SMCD19) showed a continuous increase in predicted probability values from birth to the onset of old age. These changes were also observed when the study population was segregated by sex. Of note, an increase in eEFCD4 and a decrease in eACD4 were selectively observed in the male subgroup (Figure 6).

**FIGURE 6:**
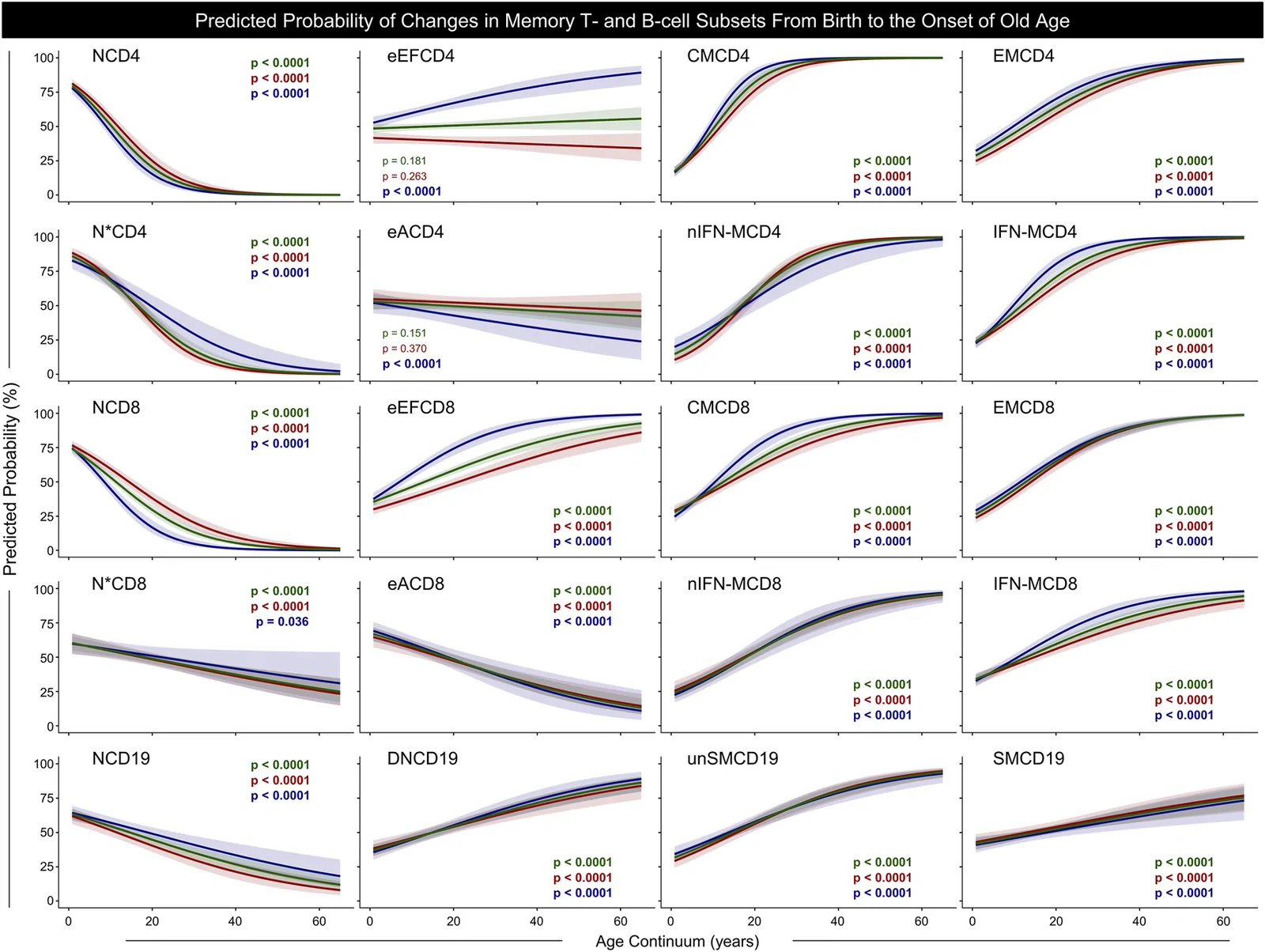
Predicted probability of changes in memory T- and B-cell subsets from birth to the onset of old age and according to sex. The association between changes in memory T-and B-cell subsets with age continuum (study population/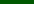, n = 1,796), further categorized according to sex (female/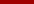, n = 926 and male/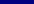, n = 870). Data categorization was performed based on cut-offs established at the 50th percentile of the empirical distribution of each dataset. The predicted probabilities of subjects to present a given memory phenotype were calculated by logistic regression to assess the association between memory T- and B-cell subsets with age continuum, as described in the *Materials and methods* section. Predicted probabilities were estimated by age, and the final data are shown as lines representing the changes in memory T- and B-cell subsets along age continuum (years) and significant differences considered at p < 0.05. The p-values for the study population and data stratification by sex (female and male) are provided in the figure. Significant p-values are underscored in bold format.

### Panoramic signatures of memory T- and B-cell subsets along age ranges and sexes

The panoramic profile of immune memory cell subsets was further characterized using the concept of overall signatures of T- and B-cell subsets. Data initially expressed as the percentage of gated cells were converted into categorical data to estimate the proportion of subjects above the global median cut-off established for each cell subset (Figure 7). The percentage of high producers of T- and B-cell subsets showed two distinct patterns. The number of individuals above the median progressively decreased across age ranges in naïve subsets (NCD4, N*CD4, NCD8, N*CD8, and NCD19) and early activated cells (eACD4 and eACD8), while it increases progressively across age ranges in eEFCD4, CMCD4, EMCD4, nIFN-MCD4, IFN-MCD4, eEFCD8, CMCD8, EMCD8, nIFN-MCD8, IFN-MCD8, DNCD19, unSMCD19, and SMCD19 in the study population, female group, and male group. Overall, the turning point for high producers of T- and B-cell subsets was the age range of 10–19 years, with a reduction in naïve subsets (<50% of high producers) and an elevation in other cell subsets (>50% of high producers) (Figure 7A).

**FIGURE 7:**
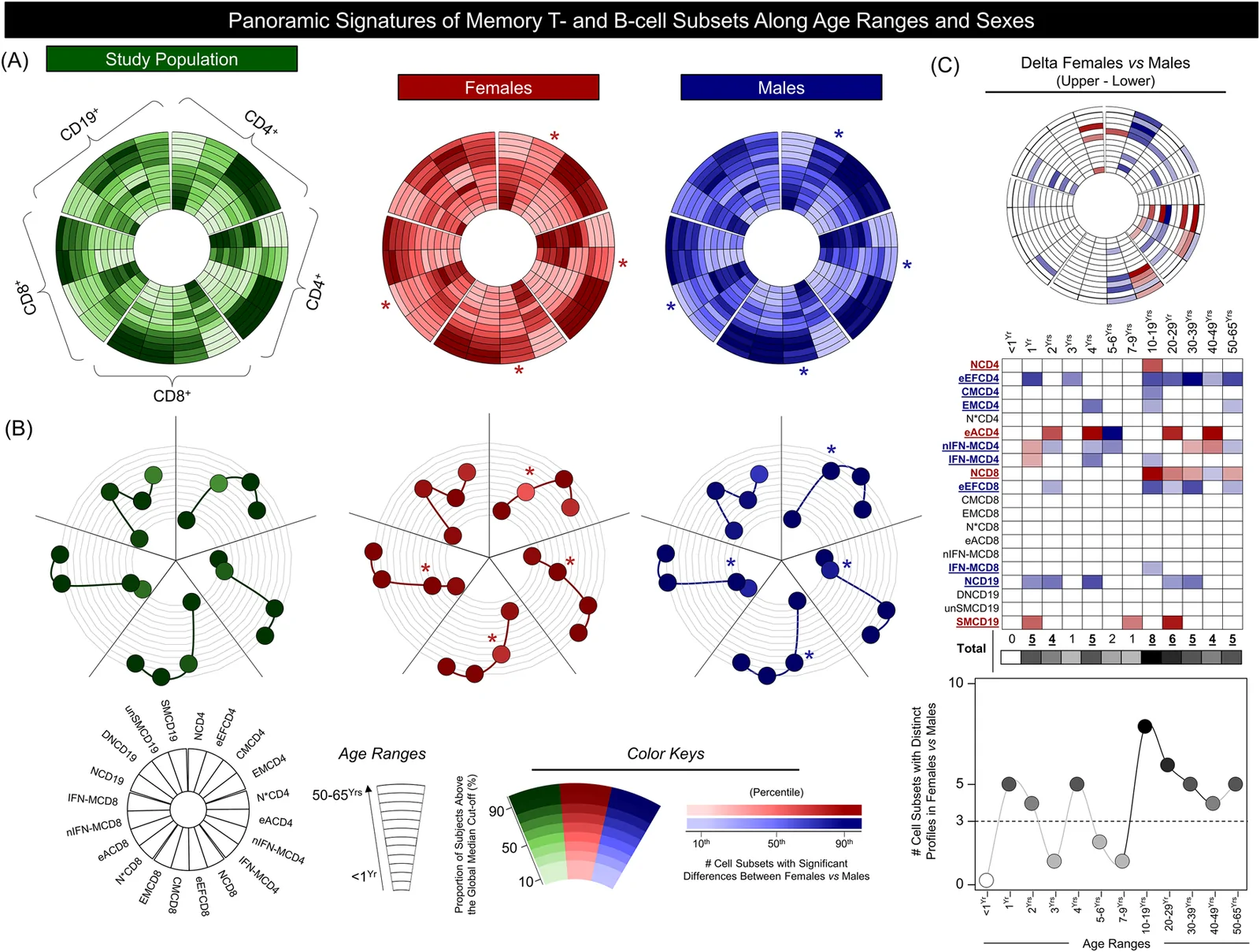
Panoramic signatures of memory T- and B-cell subsets along age ranges and sexes. The overall signature of memory T- (CD4^+^ and CD8^+^) and B-cell subsets was characterized in PBMC from healthy individuals (study population/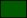, n = 1,796), categorized according to sex (female/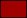, n = 926; male/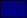, n = 870) and followed by stratification into distinct age ranges (<1 year, n = 119, n = 96; 1 year, n = 29, n = 21; 2 years, n = 100, n = 89; 3 years, n = 69, n = 79; 4 years, n = 25, n = 46; 5–6 years, n = 83, n = 81; 7–9 years, n = 104, n = 105; 10–19 years, n = 111, n = 209; 20–29 years, n = 98, n = 24; 30–39 years, n = 102, n = 67; 40–49 years, n = 60, n = 39; and 50–65 years, n = 33, n = 14, respectively). Immunophenotypic analyses of naïve (N), early effector (eEF), central memory (CM), effector memory (EM), naïve* (N*), early activated (eA), non-interferon-mediated (nIFN-M), and interferon-mediated (IFN-M) T-cells, as well as naïve (N), double-negative (DN), unswitched memory (unSM), and class-switched memory (SM) B-cells were carried out using flow cytometry as described in the *Materials and methods* section. Signatures were assembled by first converting the original data, expressed as continuous variables (% of gated cells), into categorical results reported as proportion (%) of subjects with results above the cut-off edges (global median values of each cell subset). **(A)** The final data are shown in a circular sunburst diagram plot. **(B)** In addition, the peak proportion for each age-range is displayed as an orbital plot. **(C)** The difference (delta) between signature values was calculated as the highest minus the lowest value and visualized using colormaps and line graphs. The scales and color keys are provided in the figure. Cell phenotypes with statistical differences between sexes are highlighted with *.

Consistent with the sex-related dynamics described in previous analysis, the maximum signature value differs between sexes for early effector (eEF) and early activated (eA) T-cell subsets, with early activated cell subsets occurring at earlier age ranges in the male group and early effector cell subsets in the female group (Figure 7B).

In line of the results presented in Figure 3, in four cell subsets (NCD4, eACD4, NCD8, and SMCD19), the signature is higher in the female group, and in eight cell subsets (eEFCD4, CMCD4, EMCD4, nIFN-MCD4, IFN-MCD4, eEFCD8, IFN-MCD8, and NCD19), the signature is higher in the male group. Nevertheless, the profile showed high variability, with a peak at 10–19 years (Figure 7C).

### Integrative network structures of memory T- and B-cell subsets from birth to the onset of old age

A more complete understanding of the rhythmic profile of immune memory cell subsets throughout aging was pursued by creating integrative networks of correlations between T- and B-cell subsets to assess their neighborhood connectivity (Figure 8). Network topology revealed slight loss in correlation density with age (Figure 8A). Analysis of the integrative network shows a wave-like profile of total connections, with a simple peak at 2 years and a long-lasting peak from 5 years to 19 years, followed by a continuous decrease in connectivity until 65 years. A similar profile was observed for naïve cell subsets, while memory T-cells presented a comparable rhythmic pattern, but with a more prominent reduction in the number of connections. In contrast, memory B-cells presented an increasing profile throughout aging, with a peak from 5 years to 19 years (Figure 8B).

**FIGURE 8:**
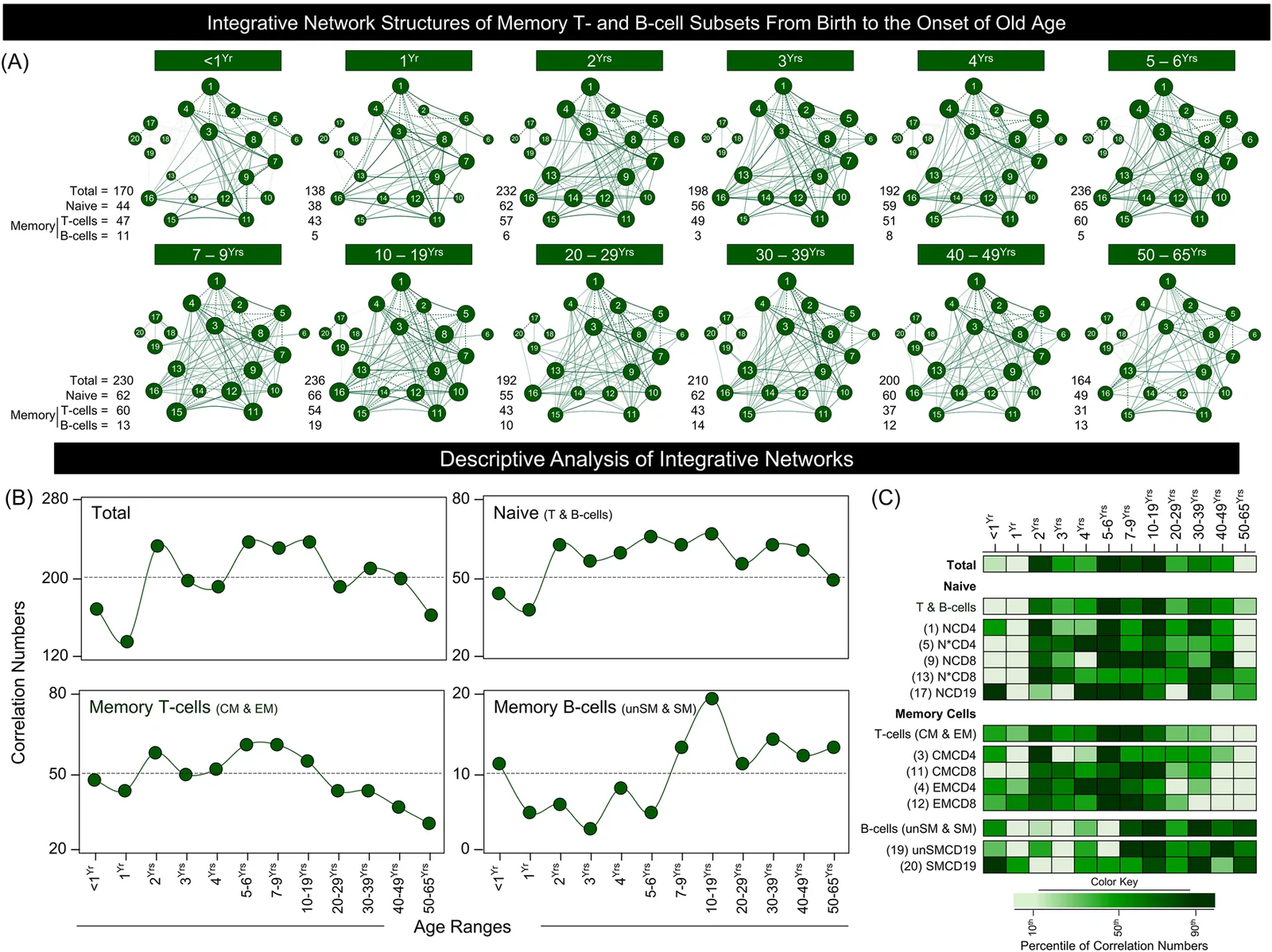
Integrative network structures of memory T- and B-cell subsets from birth to the onset of old age. Networks were constructed for memory T- (CD4^+^ and CD8^+^) and B-cell subsets from healthy individuals (study population/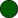, n = 1,796), categorized into distinct age ranges (<1 year, n = 208; 1 year, n = 50; 2 years, n = 189; 3 years, n = 148; 4 years, n = 71; 5–6 years, n = 164; 7–9 years, n = 209; 10–19 years, n = 320; 20–29 years, n = 122; 30–39 years, n = 169; 40–49 years, n = 99; and 50–65 years, n = 47). Immunophenotypic analyses of NCD4 (1), eEFCD4 (2), CMCD4 (3), EMCD4 (4), N*CD4 (5), eACD4 (6), nIFN-MCD4 (7), IFN-MCD4 (8), NCD8 (9), eEFCD8 (10), CMCD8 (11), EMCD8 (12), N*CD8 (13), eACD8 (14), nIFN-MCD8 (15), IFN-MCD8 (16), NCD19 (17), DNCD19 (18), unSMCD19 (19), and SMCD19 (20) were carried out by flow cytometry, as described in the *Materials and methods* section. **(A)** Networks were constructed using cluster layouts comprising five groups of memory cell phenotypes, namely, CD4^+^, CD4^+^-interferon, CD8^+^, CD8^+^-interferon, and CD19^+^ subsets. The continuous line corresponds to positive correlations, and the dashed line corresponds to negative correlations. The line thickness illustrates the correlation strength, comprising weak/moderate (“r” scores from |0.1 to 0.67|, thin lines) and strong correlations (“r” scores from ≥ |0.68|, thick lines), according to Akoglu, (2018). The node sizes are proportional to the number of correlations between cell subsets, and the axes shared between age ranges are highlighted. The total number of correlations is provided in the figure. **(B)** A descriptive analysis was performed using line graphs, considering the total number of correlations and the number of correlations of naive T- and B-cells, memory T-cells (CM and EM), and memory B-cells (unSM and SM). **(C)** A color map is created to display the number of connections for each cell cluster and each highlighted cell subset. The color key is provided in the figure.

Despite the different trends observed for memory T-cell and B-cell subsets, the magnitude of changes was more pronounced in the T-cell subsets. In addition, the age of 5–19 years marks the trend of changes. Another point that stands out is the predominance of memory T-cell connectivity compared to memory B-cell connectivity, a characteristic that is not age-dependent (Figure 8C).

### Neighborhood connectivity structures of memory T- and B-cell subsets from birth to the onset of old age according to sex

A complementary view of interplay between the subsets of immunological memory cells was performed through correlation analysis according to sex, and the resulting profiles are presented in Figure 9. The network topology revealed a significant loss in correlation density with age in the male group, while in the female group, the correlation density remains relatively stable throughout life (Figure 9A).

**FIGURE 9:**
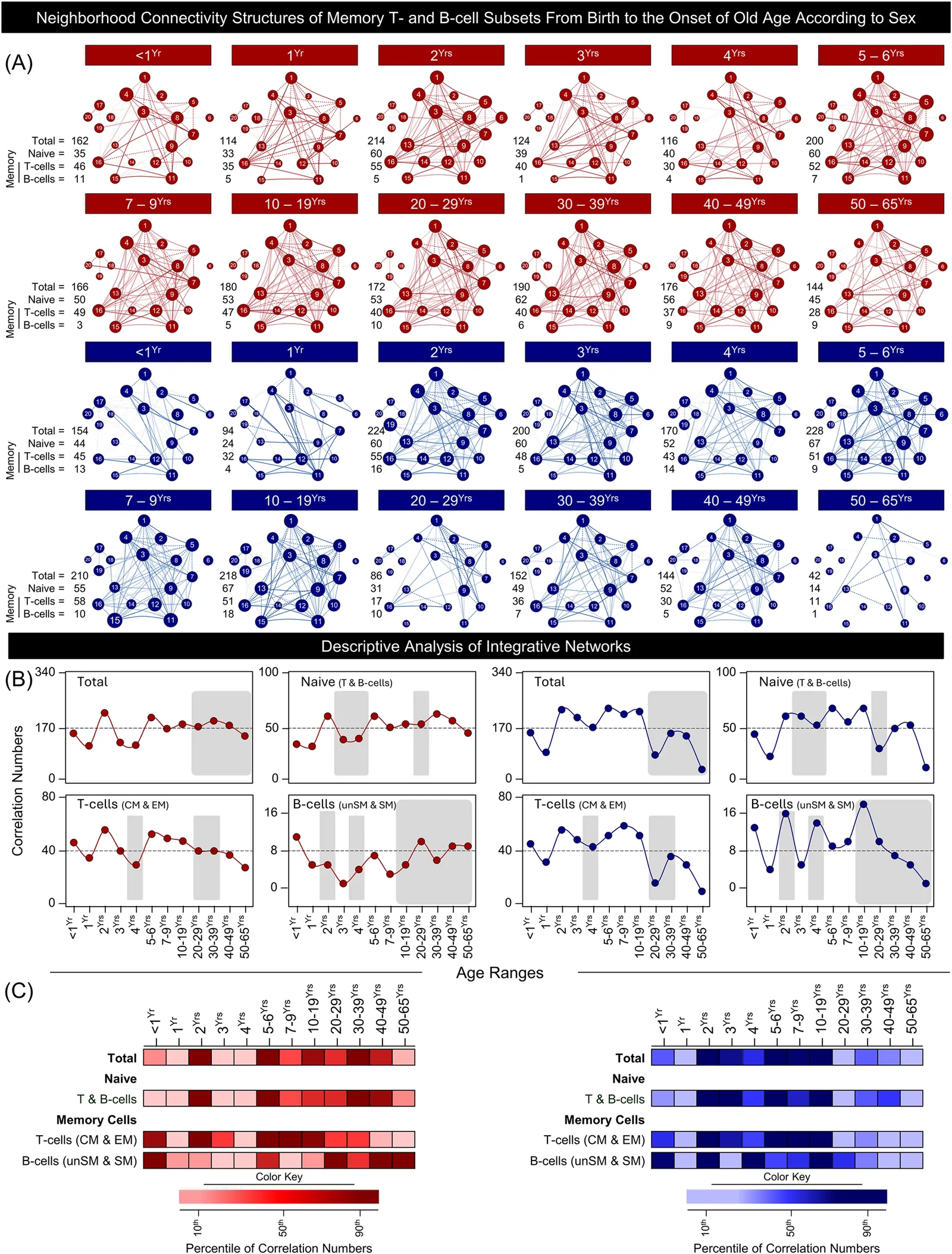
Neighborhood connectivity structures of memory T- and B-cell subsets from birth to the onset of old age according to sex. Comprehensive networks were constructed for memory T- (CD4^+^ and CD8^+^) and B-cell subsets, characterized in PBMC from healthy individuals (n = 1,796), categorized according to sex (female/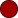, n = 926; male/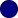, n = 870) and followed by stratification into distinct age ranges (<1 year, n = 119, n = 96; 1 year, n = 29, n = 21; 2 years, n = 100, n = 89; 3 years, n = 69, n = 79; 4 years, n = 25, n = 46; 5–6 years, n = 83, n = 81; 7–9 years, n = 104, n = 105; 10–19 years, n = 111, n = 209; 20–29 years, n = 98, n = 24; 30–39 years, n = 102, n = 67; 40–49 years, n = 60, n = 39; and 50–65 years, n = 33, n = 14, respectively). Immunophenotypic analyses of NCD4 (1), eEFCD4 (2), CMCD4 (3), EMCD4 (4), N*CD4 (5), eACD4 (6), nIFN-MCD4 (7), IFN-MCD4 (8), NCD8 (9), eEFCD8 (10), CMCD8 (11), EMCD8 (12), N*CD8 (13), eACD8 (14), nIFN-MCD8 (15), IFN-MCD8 (16), NCD19 (17), DNCD19 (18), unSMCD19 (19), SMCD19 (20) were carried out using flow cytometry, as described in the *Material and methods* section. **(A)** Networks were constructed using cluster layouts comprising five groups of memory cell phenotypes, namely, CD4^+^, CD4^+^-interferon, CD8^+^, CD8^+^-interferon, and CD19^+^ subsets. The continuous line corresponds to positive correlations, and the dashed line corresponds to negative correlations. The line thickness illustrates the correlation strength, comprising weak/moderate (“r” scores from |0.1 to 0.67|, thin lines) and strong correlations (“r” scores from ≥ |0.68|, thick lines), according to Akoglu, (2018). The node sizes are proportional to the number of correlations between cell subsets, and the axes shared between age ranges are highlighted. The total number of correlations is provided in the figure. **(B)** A descriptive analysis was performed using line graphs, considering the total number of correlations and the number of correlations of naive T-cells and B-cells, memory T-cells (CM and EM), and memory B-cells (unSM and SM). The major differences between sexes were underscored by grey background (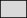). **(C)** A color map is created to display the number of connections for each cell cluster and each highlighted cell subset. The color keys are provided in the figure.

Data analysis shows the wave-like profile of total connections for female and male groups; however, the differences became more pronounced from 20 years onward, with a significant decrease in the correlation numbers in the male group. In the correlation numbers of naïve subsets, the differences between sexes were notable from 3 to 4 years and from 20 to 29 years. The memory T-cell subsets presented differences in the correlation numbers at 4 years and from 20 to 39 years. Memory B-cell subsets showed the greatest differences, initially at 2 and 4 years, followed by opposite trends from 10 years onward, with a significant decrease in the correlation numbers in the male group, while in the female group, the correlation numbers increased (Figures 9B,C). The most connected cell subsets in both groups were NCD4, NCD8, CMCD4, N*CD4, and IFN-MCD4 (Figure 9A).

### Number of correlation axes of memory T- and B-cell subsets from birth to the onset of old age according to sex

Aiming to construct a reinforcement analysis, correlation axes were explored based on neighborhood connectivity structures, according to sex. The axes were defined from the shared correlation between adjacent age ranges (Figure 10). Although the shared connections were concentrate from 5 to 49 years in the female group, the axes in the male group were concentrated from 2 to 19 years. These findings indicate distinct connectivity dynamics between sexes, with correlation axes in the female group emerging later but displaying considerable persistence and longer sharing of connections across the lifespan (Figure 10).

**FIGURE 10:**
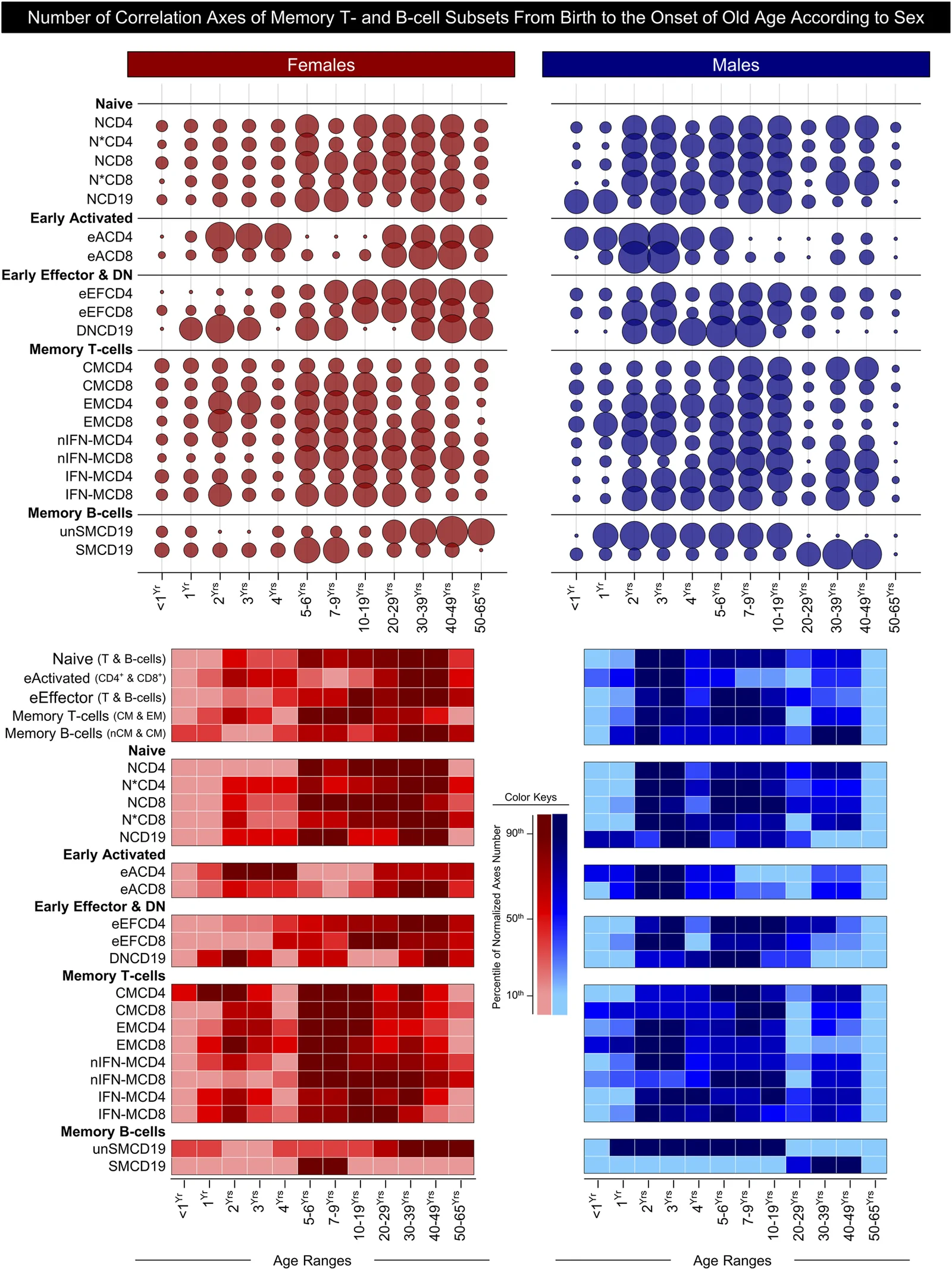
Number of correlation axes of memory T- and B-cell subsets from birth to the onset of old age according to sex. The sexes orbits were built for memory T- (CD4^+^ and CD8^+^) and B-cell subsets from healthy individuals (n = 1,796), categorized according to sex (female/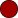, n = 926; male/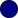, n = 870) and followed by stratification into distinct age ranges (<1 year, n = 119, n = 96; 1 year, n = 29, n = 21; 2 years, n = 100, n = 89; 3 years, n = 69, n = 79; 4 years, n = 25, n = 46; 5–6 years, n = 83, n = 81; 7–9 years, n = 104, n = 105; 10–19 years, n = 111, n = 209; 20–29 years, n = 98, n = 24; 30–39 years, n = 102, n = 67; 40–49 years, n = 60, n = 39; and 50–65 years, n = 33, n = 14, respectively). Immunophenotypic analyses of naïve (N), early effector (eEF), central memory (CM), effector memory (EM), naïve* (N*), early activated (eA), non-interferon-mediated (nIFN-M), and interferon-mediated (IFN-M) T-cells, as well as naïve (N), double-negative (DN), unswitched memory (unSM), and class-switched memory (SM) B-cells were carried out using flow cytometry, as described in the *Materials and methods* section. The analysis of correlations shared between age ranges provided axes. The final data are shown in the abacus chart, with each circle representing a single memory cell subset with size proportional to the number of axis connections. A color map is created to display the number of connections for each cell cluster and each cell subset. The color keys are provided in the figure.

## Discussion

Physiological aging is characterized by extensive and progressive remodeling of the immune system, tightly synchronized with ontogenetic alterations in memory T- and B-cell network architecture throughout the lifespan. Several studies have employed *ex vivo* approaches to perform analyses of memory T- and B-cell populations. The major contribution of the present study is to provide evidence about the dynamic remodeling of immune system along aging in a geographic area considered hyperendemic for several parasitic, viral, and infectious diseases. Previous studies from our group have shown that living in an endemic area for infectious diseases is associated to differences in immunosenescence and inflammatory signatures (Coelho et al., 2025). Moreover, living in endemic areas for parasitic and infectious diseases results in accelerated remodeling of inflammaging and leads to epigenetic aging regardless of the infectious status (Durso et al., 2022). It has been proposed that in endemic countries, biological age may exceed chronological age due to high exposure to parasitic and infectious diseases. A plausible mechanism underlying this phenomenon is that constant exposure to parasites or viruses may cause immune exhaustion. In this sense, it is expected that subjects living in hyperendemic areas for parasitic/infectious diseases exhibit a dissociation between chronological age and biological aging measured by DNA methylation. The immunological events associated with the biological/chronological aging dissociation have been partially investigated and seem to be mediated by a unique immunological profile (Comin et al., 2008, Torres et al., 2018, Silveira-Nunes et al., 2017, Batista et al., 2020). In endemic areas, non-infected subjects may develop compensatory mechanisms to cope with immune dysfunction observed in infected individuals. Two mechanisms have been proposed to contribute to this phenomenon. First, non-infected subjects develop innate immune responses to overcome the decrease in T-cell function observed with aging. Second, infected aged individuals present more vigorous regulatory T-cells that may impair their immune response (Comin et al., 2008). Therefore, aging does not affect all subjects homogeneously, and the resulting memory phenotypes seems to be determined by genetic, epigenetic, and immunobiographic factors affected by interactions between the immune system, infectious disease history and environmental features.

In the present study, the use of long-term cultures of PBMCs offers a robust alternative for evaluating distinct memory phenotypes, including central (TCM) and effector (TEM) memory subsets. Methodologically, extended culture periods allow cells to recover from isolation stress, undergo clonal expansion, and lineage differentiation, thereby providing a more accurate reflection of their functional kinetics and homeostatic behavior over time. The latter approach has been selected in the present study as the rational to investigate T- and B-cell memory phenotypes. *In vitro* culturing contributes to PBMC recovery from isolation stress, restoring the homeostatic behavior. Using this approach, our results demonstrated that immune memory remodeling across the human lifespan follows discrete, sex-dependent trajectories, reflecting structured immunobiographical imprinting of adaptive immunity from early life to early aging. The results indicate that immune remodeling that shapes adaptive immunity does not proceed linearly but rather occurs in waves, with an earlier onset in women but greater amplitude of differentiation in men, particularly within effector memory subsets. Divergent connectivity patterns further suggest that female and male groups adopt distinct strategies of immune network organization; male subjects experience a significant decrease in connectivity in 20–29 years and 50–65 years, whereas female subjects exhibit sustained high connectivity throughout aging, shaping long-term inflammatory tone and adaptive reserve essential to adaptive immunity assembly.

Within the framework of immunobiography, the combined use of antibodies against CD27/CD45RO for T-cells and CD27/IgD for B-cells provides a structured readout of how cumulative antigenic exposures across life progressively reshape the balance between naïve, double-negative, unswitched, switched, and differentiated memory phenotypes. In T-cells, CD27^+^CD45RO^−^ populations define the naïve compartment, while sequential transitions toward CD27^+^CD45RO^+^ central memory and CD27^−^CD45RO^+^ effector memory subsets reflect increasing antigenic experience and functional specialization of adaptive immunity; CD27^−^CD45RO^−^ early effector cells represent a transient and unstable differentiation state, particularly evident in children and adolescents (Brito-de-Sousa et al., 2023; Schiött et al., 2004). The incorporation of CXCR3 (CD183) further refines this immunobiographical trace of adaptive immunity by distinguishing Th1/Tc1-polarized inflammatory memory cells (CD183^+^CD45RO^+^), typically shaped by viral and intracellular pathogens and prone to accumulation under repeated stimulation and chronic inflammation, from non-Th1 memory trajectories (CD183^−^CD45RO^+^) associated with barrier immunity, allergy, and B-cell help (Payne et al., 2020; Krummey et al., 2020; Künzli and Masopust, 2023). These shifts likely reflect a functional trade-off in aging immunity, whereby expanded memory compartments enhance recall responses while simultaneously increasing basal inflammatory tone (Franceschi et al., 2018).

When this conceptual framework is applied to age-stratified quantitative analyses, immune remodeling emerges as a temporally structured and sex-dependent process rather than a linear, continuous trajectory. Statistically significant changes between adjacent age ranges are concentrated in specific intermediate windows, with an early transition between 5–6 years, followed by a major remodeling phase spanning until late childhood through adolescence (approximately 10–19 years). Subsequently, a more discrete wave of changes occurs during early adulthood (20–29 years). Outside these windows, adjacent age groups display comparatively fewer significant differences, suggesting relative stabilization phases in immune composition. This pattern aligns with the notion that immunobiographical imprints of adaptive immunity are preferentially established during the periods of intense antigenic exposure and immune network reorganization, rather than accumulating uniformly over time.

Across multiple T-cell phenotypes, men consistently exhibit higher median values than women, with statistically significant sex differences observed specially for eEFCD4. Additionally, other subsets also presented higher median values in men, such as CMCD4, EMCD4, IFN-nMCD4, IFN-MCD4, eEFCD8, and IFN-MCD8, particularly between 10 and 39 years. These findings suggest an accumulation or persistence of differentiated and inflammatory-leaning T-cell subsets in male individuals during the late childhood, adolescence, and early adulthood. Notably, cumulative fold-change analyses reveal that although immune remodeling in women begins earlier, men display larger magnitude of variations across the lifespan, with the most pronounced effects involving eEF populations that are essential to build up adaptive immunity. Signature-based analyses corroborate this interpretation, demonstrating that peaks in the proportion of individuals exceeding the global median occur at distinct ages in men and women, with consistently higher signature values in men for the same T-cell subsets. Together, these results suggest that male immunobiography is characterized not by earlier onset but by greater amplitude of differentiation and inflammatory polarization.

These quantitative patterns integrate well with established age-related remodeling of the T-cell compartment that underpins adaptive immunity. In infancy and childhood, peripheral blood T-cells are dominated by the naïve CD27^+^CD45RO^−^ subset, reflecting high thymic output and limited antigenic experience (Cossarizza et al., 1996; van Gent et al., 2009). With aging, thymic involution and repeated antigen exposure drive a progressive contraction of this compartment, particularly within CD8^+^ T-cells (Li et al., 2019; Kulesh et al., 2025; Saule et al., 2006). Loss of naïve cells is more pronounced in CD8^+^ than in CD4^+^ T-cells, leading to early dominance of effector memory and its variants (Rodriguez et al., 2021; Koch et al., 2008; Jain et al., 2023). These populations frequently express senescence-associated markers such as CD57 and KLRG1 and often show clonal expansion in response to persistent viral infections, functionally linking them to immunosenescence and inflammaging through reduced proliferative capacity and enhanced production of pro-inflammatory cytokines such as IL-6, IL-1α, IL-1β, IL-8, and TNF (Li et al., 2019; Ventura et al., 2017; Smetanova et al., 2022).

In contrast, CD4^+^ T-cells display a slower decline in the naïve subset, with memory accumulation occurring predominantly through the expansion of central memory (CD27^+^CD45RO^+^) and effector memory (CD27^−^CD45RO^+^) subsets (Saule et al., 2006; Heikkilä et al., 2022). Terminally differentiated CD27^−^CD45RO^−^ CD4^+^ cells remain comparatively infrequent even in advanced age (Koch et al., 2008; Jain et al., 2023). Nevertheless, aging CD4^+^ memory T-cells undergo marked functional remodeling, including mitochondrial accumulation, increased reactive oxygen species production, and heightened secretion of pro-inflammatory mediators such as IFN-γ, IP-10, and IL-6, reinforcing their contribution to systemic inflammaging (Mittelbrunn and Kroemer, 2021; Chen et al., 2022).

Differentiation within the T-cell compartment follows continuous phenotypic gradients rather than discrete states. Loss of CD27 and CD28 marks progressive differentiation, while acquisition of CD45RO reflects memory status (Rodriguez et al., 2021; Koch et al., 2008). Expression of CXCR3 further delineates functional trajectories: CD27^+^CD183^−^CD45RO^−^ cells represent minimally differentiated, naïve-like populations abundant in childhood but steadily decreasing with age, whereas CD183^+^CD45RO^−^ cells retain a naïve surface phenotype yet exhibit Th1-like inflammatory priming, increasing in relative frequency with aging due to homeostatic proliferation under chronic inflammatory conditioning (van Gent et al., 2009; Teteloshvili et al., 2015; Yoshida et al., 2024). Fully differentiated CD183^+^CD45RO^+^ memory T-cells expand markedly from childhood to adulthood and persist at high frequency in older individuals, often displaying clonal expansion and pro-inflammatory polarization, thereby shaping adaptive immunity (Cossarizza et al., 1996; Britanova et al., 2016; Wang et al., 2025).

Parallel to T-cell remodeling, the B-cell compartment undergoes age-dependent restructuring that is strongly sex-specific when viewed through network connectivity analyses. In early life, peripheral blood B-cells are dominated by naïve CD19^+^CD27^−^IgD^+^ cells, reflecting limited antigen exposure and ongoing maturation of humoral adaptive immunity (van Gent et al., 2009; Mandala and Longwe, 2021). During childhood and adolescence, progressive antigen encounter drives expansion of memory subsets, including non-switched (CD27^+^IgD^+^) and class-switched (CD27^+^IgD^−^) memory B-cells generated through germinal center reactions (Caraux et al., 2010; Muggen et al., 2019).

In healthy adults, naïve B-cells often remain the largest subset, but memory populations constitute a substantial fraction of the repertoire (Nevalainen et al., 2019; Chayé et al., 2025). With aging, absolute numbers of CD19^+^ B-cells decrease, accompanied by disproportionate loss of class-switched memory B-cells and plasmablasts (Caraux et al., 2010; Muggen et al., 2019; Feng et al., 2022). In parallel, unswitched memory populations and double-negative (CD27^−^IgD^-^) B-cells expand in relative or absolute terms and are associated with elevated IL-6 levels, frailty, autoimmunity, and chronic inflammatory states, particularly in men (Nevalainen et al., 2019; Cancro, 2020; Li et al., 2021; Chung et al., 2023). Consistent with these observations, our findings indicate age-dependent modulation of memory B-cell subsets, including double-negative B-cells. Although our cohort consisted of clinically healthy individuals and no autoimmune or chronic inflammatory conditions were identified at the time of sampling, these changes do not necessarily imply ongoing pathology. Rather, they may reflect immunobiographical trajectories that, under additional genetic or environmental pressures, could influence susceptibility to inflammatory or autoimmune diseases later in life.

Network-level analyses reveal that naïve cell subsets exhibit the highest number of connections, underscoring their central role in maintaining immune network architecture. However, profound sex differences emerge in the B-cell compartment: B-cell connectivity increases progressively with age in women, whereas in men, it decreases sharply, resulting in a marked reduction in total network connectivity from approximately 20 years of age onward. Unlike the male individuals, connectivity among the female individuals remains elevated throughout their lives, with less apparent variations. Consistently, connectivity axes arise earlier in men (approximately 2–19 years) but are more intense and prolonged in women, extending from 5 to 49 years. Importantly, upregulation of cell subtypes mirrors global connectivity patterns, indicating that phenotypic diversification and network integration are tightly coupled processes.

It is important to mention that the present work has some limitations. Although the study was designed as a multicenter investigation comprising a non-probabilistic convenience sampling with strict inclusion and exclusion criteria adopted to minimize major sources of bias, data analysis was not adjusted for additional factors, such as lifetime history of infectious/parasitic exposures, cumulative vaccination exposure, nutritional status, endocrine transitions, and environmental influences that could interfere in the exploratory investigation of immunobiographical profiles. These variables are recognized as interferents of immune remodeling and may have contributed to the interindividual variability observed in the present study. However, within the framework of immunobiography, such factors should not be viewed solely as confounders but also as intrinsic components of the cumulative biological experiences that shape immune memory throughout life. Consequently, part of the heterogeneity observed among clinically healthy individuals may reflect physiological differences in immunobiographical trajectories rather than chronological age alone. In this regard, longitudinal studies integrating immunophenotyping with metabolic, endocrine, infectious, and environmental data represent a promising field for future research aimed at a deeper understanding of the impact of these factors on immunobiography. Another limitation that should be considered is that the observational nature of this investigation and the distinct sample sizes across age groups would impact the findings. Additionally, the present study has focused on the characterization of changes in phenotypic features of memory T- and B-cell subsets and did not include a simultaneous investigation of systemic soluble immune mediators using the same sample, which should be addressed in further investigations to provide a more meaningful assessment of age-related immune alterations.

Taken together, these findings support a model in which adaptive immunity aging reflects the dynamic integration of antigenic history, sex-specific endocrine modulation, and evolving immune network architecture. Early-life and pubertal windows emerge as critical periods within the immunobiographical trajectories, while adulthood and senescence are characterized by differential amplification, stabilization, or collapse of these networks. Within this framework, the balance between central and late effector memory in both T-cells and B-cells represents a key determinant of immune flexibility versus inflammatory burden, linking cellular phenotypes, network connectivity, and lifelong immune health.

## Data Availability

The raw data supporting the conclusions of this article will be made available by the authors, without undue reservation.
